# Categorization of collagen type I and II blend hydrogel using multipolarization SHG imaging with ResNet regression

**DOI:** 10.1038/s41598-023-46417-0

**Published:** 2023-11-09

**Authors:** Anupama Nair, Chun-Yu Lin, Feng-Chun Hsu, Ta-Hsiang Wong, Shu-Chun Chuang, Yi-Shan Lin, Chung-Hwan Chen, Paul Campagnola, Chi-Hsiang Lien, Shean-Jen Chen

**Affiliations:** 1 College of Photonics, National Yang Ming Chiao Tung University, Tainan, Taiwan; 2 Department of Medical Education, National Taiwan University Hospital, Taipei, Taiwan; 3 Orthopaedic Research Center, Kaohsiung Medical University, Kaohsiung, Taiwan; 4 Department of Orthopedics, College of Medicine, Kaohsiung Medical University, Kaohsiung, Taiwan; 5 Department of Biomedical Engineering, College of Engineering, University of Wisconsin-Madison, Madison, WI USA; 6 Department of Mechanical Engineering, National United University, Miaoli, Taiwan

**Keywords:** Biotechnology, Optics and photonics

## Abstract

Previously, the discrimination of collagen types I and II was successfully achieved using peptide pitch angle and anisotropic parameter methods. However, these methods require fitting polarization second harmonic generation (SHG) pixel-wise information into generic mathematical models, revealing inconsistencies in categorizing collagen type I and II blend hydrogels. In this study, a ResNet approach based on multipolarization SHG imaging is proposed for the categorization and regression of collagen type I and II blend hydrogels at 0%, 25%, 50%, 75%, and 100% type II, without the need for prior time-consuming model fitting. A ResNet model, pretrained on 18 progressive polarization SHG images at 10° intervals for each percentage, categorizes the five blended collagen hydrogels with a mean absolute error (MAE) of 0.021, while the model pretrained on nonpolarization images exhibited 0.083 MAE. Moreover, the pretrained models can also generally regress the blend hydrogels at 20%, 40%, 60%, and 80% type II. In conclusion, the multipolarization SHG image-based ResNet analysis demonstrates the potential for an automated approach using deep learning to extract valuable information from the collagen matrix.

## Introduction

Osteoarthritis, a degenerative joint disease, is associated with alterations in the collagen composition within the cartilage matrix^1^. Investigating collagen type I and II mixtures in osteoarthritis can provide insights into the underlying mechanisms of cartilage degeneration. The dynamic interrelationship among type I and type II collagen, in conjunction with other elements of the matrix and cellular functions, exerts a significant impact on maintaining the delicate equilibrium between the degradation and reparative processes within cartilages^2^. Second harmonic generation (SHG) microscopy is a powerful technique for visualizing 3D collagen fibers with exceptional resolution^3^. It utilizes the non-centrosymmetric molecular arrangements in biological tissues, such as collagen, myosin, tendons, and muscles, to generate second harmonic signals^4,5^. By employing polarization-resolved SHG (P-SHG) imaging, researchers can obtain detailed quantitative insights into sub-cellular collagen structures^6^. P-SHG imaging has gained significant interest in various medical disciplines, including breast cancer diagnosis^6^, skin irregularities assessment^7^, muscle characterization^8^, osteoarthritis diagnosis^9^, and detection of diseased tissues^10^. It allows for differentiation capabilities at the fiber-level collagen structures, providing valuable information for medical research and diagnostics. Various methods can be used for quantification and analysis of P-SHG specimens, including geometrical and statistical analysis, Fourier transform-based analysis, phasor approach, and grey-level co-occurrence matrix-based texture analysis. P-SHG imaging is particularly adept at capturing microscopic structural information with high spatial resolution and it directly visualizes the anisotropic nature of materials, revealing the orientation and alignment of molecular structures. The dipole moment of collagen is represented by a power series that includes the first hyperpolarizability term and this term describes the interaction of collagen with the incident electric field. Overall, P-SHG imaging offers unparalleled contrast and specificity in visualizing detailed collagen structures in tissues, making it an indispensable tool for understanding complex biological processes and advancing medical diagnostics^11,12^. However, P-SHG analysis for discriminating collagen types such as types I and II often stands on pixel-by-pixel calculation and involves manual efforts in data processing, computation time, parameter extraction, and interpretation of results. This subjectivity can introduce bias and may require domain expertise, making it less automated and potentially more time-consuming.

Deep learning (DL) has revolutionized various domains in computer vision, achieving remarkable success in image classification, object detection, and segmentation tasks^13,14^. DL models, although complex, can be more interpretable with appropriate techniques, such as visualization of feature maps, and can also be trained to handle noise and variations in data, making them more adaptable to real-world scenarios. Conventional P-SHG-based analysis has its strengths in investigating specific physical properties; however, DL analysis can offer advantages in terms of automation, generalization, scalability, and adaptability to various domains and large-scale datasets. One of the most significant advancements in the realm of convolutional neural networks (CNNs) is the introduction of ResNet (short for Residual Network) in 2015, which has gained significant popularity in various computer vision tasks, including image segmentation^15^. This architecture has since gained widespread attention for its deep learning exceptional ability to tackle the challenges of training very deep neural networks^16–18^. ResNet encourages each layer to learn the difference between the current input and the desired output, effectively learning only what is new and complex. This approach mitigates the vanishing gradient issue, allowing the model to be trained effectively even with dozens or hundreds of layers. Regression tasks involve predicting continuous numerical values, making them applicable in situations where precise quantification is crucial^19^. In the context of biomedical research and medical imaging, regression models can be particularly valuable in quantifying the properties of tissues, organs, or other biological structures^20,21^. When adapting ResNet for regression tasks, the architecture's residual blocks can be modified to suit the nature of the prediction problem. The final fully connected layers are adjusted to produce a single continuous output instead of class probabilities. By integrating polarization SHG images into the ResNet architecture for regression tasks, the benefits of both modalities can be exploited. During the training process, ResNet learns to map the input polarization SHG images to the corresponding target values, effectively learning the complex relationships between polarization patterns and the underlying sample properties.

Recently, cartilage tissue engineering has emerged as a promising field in regenerative medicine, aiming to address the challenges associated with cartilage repair and regeneration. To overcome the limitations of traditional treatments, researchers have explored the development of biomimetic scaffolds that mimic the native extracellular matrix (ECM) to support cell growth and tissue regeneration. Among these scaffolds, collagen-based hydrogels have shown great potential due to their biocompatibility, biodegradability, and similarity to the natural ECM of cartilage. Several research studies have investigated the use of collagen hydrogels containing different types of collagen, such as type I and type II, in cartilage tissue engineering applications. Kilmer et al. explores the influence of type I collagen on the development and maintenance of tissue and also highlights the potential of collagen-based scaffolds for cartilage tissue engineering and repair^22^. Another study used rheological analysis to conclude that the ratio of collagen type I to collagen type II affects the protein concentrations and collagen incorporation in the gels^23^. Some studies have also quantified the structural information of fiber networks in hydrogels using SHG tensor element ratios and fiber thickness purely from polarization-based SHG images where the model is limited to a pixelized method of assuming fiber of one-pixel width^24^. In this study, the application of progressive multipolarization SHG images incorporated in a multichannel 2D ResNet is explored for the discrimination of different collagen type I and II blend hydrogels. Herein, the multipolarization SHG images refer to the set of 18 optical cross-sectional SHG images obtained by incrementally rotating the linear polarization angle in 10° intervals across 180°. Nonpolarization SHG images are generated by averaging the intensities of the 18 linear polarization images, effectively resulting in an image devoid of any polarization change. This ensures a consistent laser polarization approach for both these images, considering the inherent random alignment of collagen fibers within the sample. The ResNet regression is trained with both the multipolarization and the nonpolarization images for premixing five hydrogels with the collagen type I and II blend hydrogel composition at (a) 0%, (b) 25%, (c) 50%, (d) 75%, and (e) 100% type II. Furthermore, this trained model is utilized to predict the blend hydrogels at 20%, 40%, 60%, and 80% type II. The experimental results confirm the efficacy of incorporating both modalities into categorization and regression analysis of collagen type I and II blend hydrogel. These findings offer valuable insights that can contribute to an enhanced understanding of collagen dynamics in the extracellular matrix (ECM) during cartilage repair.

## Experimental results and discussion

### Multipolarization images of collagen blend hydrogel

**Figure 1:**
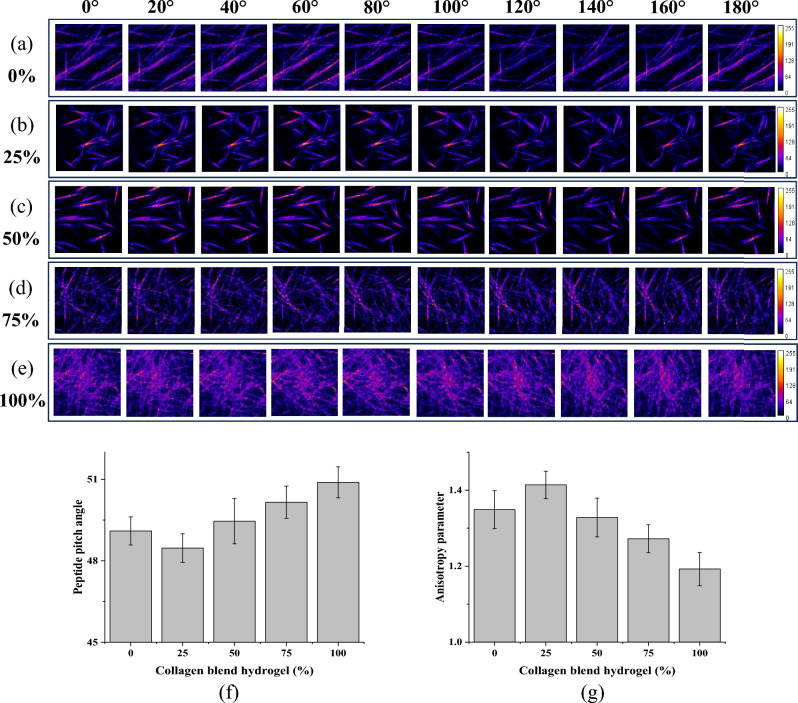
Progressive polarization images at 0°, 20°, 40°, 60°, 80°, 100°, 120°, 140°, 160°, and 180° for collagen type I and II blend hydrogels at (**a**) 0%, (**b**) 25%, (**c**) 50%, (**d**) 75%, and (**e**) 100% type II, and corresponding averaged (**f**) peptide pitch angle and (**g**) anisotropy parameter values. Error bars represent standard error of mean.

The study by Yuan et al.^25^ investigates the effects of the composition and mechanical properties of injectable collagen I/II composite hydrogels on chondrocyte behaviors in cartilage repair. The researchers observed that the microstructure and mechanical property of the hydrogels were relevant to the composition of the composite hydrogels. Hence, discriminating collagen blend gels using P-SHG images is a necessary approach to investigate and quantify the collagen type without the need for staining. Figure 1 presents a series of polarization images illustrating the variations in collagen mixture proportion achieved by incorporating different concentrations of collagen type II into collagen type I hydrogel. The images correspond to 10 different polarization degrees, representing collagen type I and II blend hydrogels at (a) 0%, (b) 25%, (c) 50%, (d) 75%, and (e) 100% type II. Figure 1f,g show the peptide pitch angle and anisotropy parameter values of the collagen type I and II blend hydrogels at 0%, 25%, 50%, 75%, and 100% type II by fitting polarization SHG pixel-wise information into generic mathematical models as mentioned earlier^11,12^^.^. The average peptide pitch angle values of the 0%, 25%, 50%, 75%, and 100% blend hydrogels are 49.01°, 48.47°, 49.46°, 50.16° and 50.89°, respectively. The average anisotropy parameter values are 1.34, 1.41, 1.33, 1.27, and 1.19, respectively. However, the average peptide pitch angle and anisotropy parameter values of the 0% blend hydrogel closely approximate those of the 50% blend hydrogel. Consequently, relying solely on the pixel-wise methods makes it challenging to discern various collagen type I and II blend hydrogels.

Notably, all the polarization images for each collagen blend proportion exhibit distinct differences in intensity as a function of polarization, facilitating the differentiation of collagen fibers. Additionally, an investigation was conducted to assess whether there were discernible differences based on fiber thickness, fiber density, or overall SHG intensity to determine whether the network utilized any of these parameters during its analysis. To ensure unbiased results, all these parameters were kept random during the study.

### Collagen blend hydrogel categorization using ResNet with SHG images

**Figure 2:**
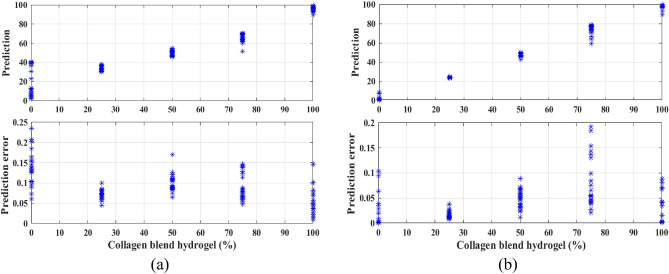
Predicted vs. actual response (top) along with the error (bottom) of prediction for collagen type I and II blend hydrogels at 0%, 25%, 50%, 75%, and 100% type II for models trained with (**a**) nonpolarization images and (**b**) multipolarization spectra as 18 channels.

Previously, the present group used pixel-wise analysis with polarization SHG information to discriminate collagen type I and type II in collagen gel assembly^11^. However, this method requires fitting polarization SHG pixel-wise information into generic mathematical models, and also reveals inconsistencies for categorizing collagen type I and II blend hydrogel. To address the limitations, the present group sought an automatic and responsive analysis method capable of efficiently differentiating collagen mixtures. Given the capacity of image-based ResNet to function without the need for prior mathematical model fitting and yield precise predictions, the conventional ResNet architecture is modified by incorporating regression and fully connected layers to predict collagen mixtures with varying percentages of collagen type I and type II based on SHG images. Figure 2a illustrates the comparison between predicted and actual responses for 5 different collagen type I and II blend hydrogels at 0%, 25%, 50%, 75%, and 100% type II, pertaining to the 2D ResNet model trained without the polarization SHG images. The figure, featuring two subplots, is designed to illustrate the relationship between collagen blend hydrogel (%) and the calculated statistical measures. The top subplot displays a scatter plot of collagen blend hydrogel (%) on the x-axis against the predicted percentages on the y-axis for each specific collagen blend hydrogel. The bottom subplot shows a scatter plot of mixed concentration on the *x*-axis against the standard deviation of each specific concentration.

Similarly, the 2D multichannel ResNet model trained on the multipolarization SHG images and the predicted vs. actual response for the collagen gel blends is as shown in Fig. 2b. For the model trained without polarization images, the error bars were *p* < 0.25 for 0% type II, *p* < 0.1 for 25% type II, *p* < 0.18 for 50% type II, *p* < 0.15 for 75% type II, and *p* < 0.15 for 100% type II. In contrast, the model trained with multipolarization images showed lower error bars with *p* < 0.12 for 0% type II, *p* < 0.05 for 25% type II, *p* < 0.1 for 50% type II, *p* < 0.2 for 75% type II, and *p* < 0.1 for 100% type II, as shown in Fig. 2b. Specifically, the *p* values for 0% and 25% type II showed an improvement of over twofold. Table 1 shows the corresponding mean difference between the predicted and actual values for the network trained on nonpolarization images (see Fig. 2a) with a mean absolute error (MAE) of 0.021 and multipolarization images (Fig. 2b) with a MAE of 0.083 for the five collagen type I and II blend hydrogels. Although the error results for multipolarization images are not significantly superior to those for nonpolarized ones, they still show improvement, possibly due to the inherent polarization properties in the images. Nevertheless, augmenting the training dataset with more images could potentially enhance the model's performance. Therefore, the examination of the error plots and prediction graphs suggested a nuanced impact on the network's categorization ability for collagen blends when utilizing polarization images compared to nonpolarization images.

**Table 1:** The mean difference between the predicted and actual values for the network trained on nonpolarization images and multipolarization images for collagen type I and II blend hydrogels at 0%, 25%, 50%, 75%, and 100% type II.

	0%	25%	50%	75%	100%
Nonpolarization	0.176 ± 0.14	0.081 ± 0.02	0.023 ± 0.01	0.093 ± 0.04	0.042 ± 0.02
Multipolarization	0.012 ± 0.02	0.013 ± 0.005	0.026 ± 0.01	0.035 ± 0.03	0.018 ± 0.02

### Collagen blend regression analysis using ResNet with SHG images

**Figure 3:**
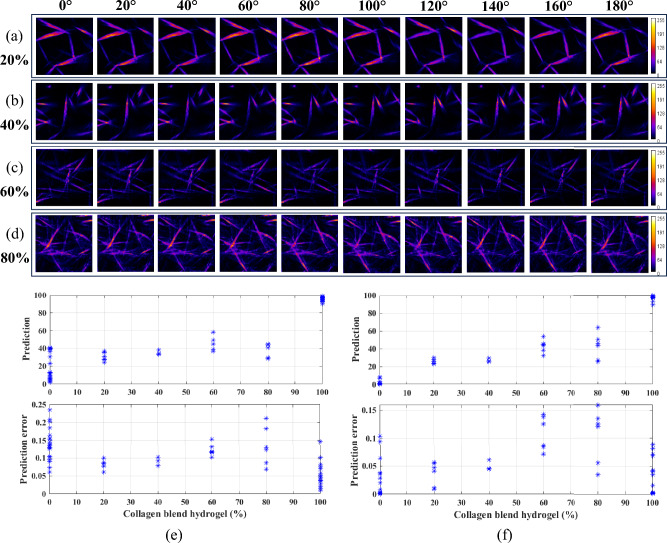
Multipolarization images at 0°, 20°, 40°, 60°, 80°, 100°, 120°, 140°, 160° and 180° for collagen type I and II blend hydrogels at (**a**) 20%, (**b**) 40%, (**c**) 60%, and (**d**) 80% type II. Predicted vs. actual response (top) along with the error (bottom) of prediction for collagen type I and II blend hydrogels at 0%, 20%, 40%, 60%, 80%, and 100% type II for models trained with (**e**) nonpolarization images and (**f**) multipolarization spectra as 18 channels.

The proposed model utilizing multipolarization images has shown successful differentiation of the collagen mixture gels at five different proportions. Furthermore, Fig. 3a–d present a series of polarization images showing the variations in collagen mixture proportions achieved by incorporating different concentrations of collagen type II into collagen type I hydrogel. The images correspond to 10 different polarization degrees, representing collagen type I and II blend hydrogels at (a) 20%, (b) 40%, (c) 60%, and (d) 80% type II. In the top subplot of Fig. 3e, the predicted versus actual responses for collagen type I and II blend hydrogels at 0%, 20%, 40%, 60%, 80%, and 100% type II are depicted for the 2D ResNet model trained without the multipolarization images. The corresponding error bars are indicated in the bottom subplot of Fig. 3e, where an error of *p* < 0.25 for 0% type II, *p* < 0.1 for 20% type II, *p* < 0.11 for 40% type II, *p* < 0.16 for 60% type II, *p* < 0.23 for 80% type II, and *p* < 0.15 for 100% type II in collagen type I hydrogel is observed. Similarly, the 2D multichannel ResNet model trained on the multipolarization SHG images and the predicted versus actual response for the collagen gel blends is shown in the top subplot of Fig. 3f. For the model trained with multipolarization images, the error bars were specifically *p* < 0.11 for 0% type II, *p* < 0.06 for 20% type II, *p* < 0.07 for 40% type II, *p* < 0.15 for 60% type II, *p* < 0.16 for 80% type II, and *p* < 0.09 for 100% type II, as shown in the bottom plot of Fig. 3f. Table 2 shows the corresponding mean difference between the predicted and actual values for the network trained on nonpolarization images (see Fig. 3e) with a MAE of 0.146 and multipolarization images (Fig. 3f) with a MAE of 0.080 for the six collagen type I and II blend hydrogels. However, it's important to note that these specific collagen blend proportions are tested using the model trained on the collagen type I and II blend hydrogels at 0%, 25%, 50%, 75%, and 100% type II. At present, correlating the collagen blend proportion prediction is challenging for the models not trained on the respective collagen blend polarization images. However, the prediction rate for the collagen blend gel still follows the trend, it can be assumed that with an increased volume of images within the training dataset, the predictions are likely to exhibit improved performance^26–28^.

**Table 2:** The mean difference between the predicted and actual values for the network trained on nonpolarization images and multipolarization images for collagen type I and II blend hydrogels at 0%, 20%, 40%, 60%, 80%, and 100% type II.

	0%	20%	40%	60%	80%	100%
Nonpolarization	0.176 ± 0.14	0.104 ± 0.04	0.047 ± 0.02	0.138 ± 0.07	0.419 ± 0.07	0.043 ± 0.02
Multipolarization	0.012 ± 0.02	0.060 ± 0.02	0.130 ± 0.02	0.170 ± 0.07	0.369 ± 0.14	0.018 ± 0.02

The polarization direction of the incident field determines the polarization direction of the emitted SHG signal and by measuring the polarization profile of the SHG signal at different incident polarization angles, the molecular structure and organization of collagen can be probed^29^. While the orientation analysis of the collagen network in three dimensions has proven effective in distinguishing healthy tissue from osteoarthritis tissue, the challenge arises from the missing substrate area available within the collagen hydrogel for maintaining collagen cohesion^30^. ResNet is designed to focus on learning only new, complex information at each layer, making it particularly well-suited for tasks involving large-scale feature extraction. Incorporating polarization images as separate channels in a multichannel ResNet allows the network to capture more diverse and informative features, which can be crucial for tasks such as material classification, object detection, and segmentation^31^. Considering polarization information allows the model to reduce ambiguity and make more accurate predictions, especially in scenarios where intensity-based features alone are insufficient^32^. 2D + 1D convolution might be more appropriate where the data can be naturally separated into spatial and spectral components, using a combination of 2D convolutions for spatial information and 1D convolutions for polarization spectral information. This approach is commonly used for processing hyperspectral data, where one has spatial information (2D) and spectral information (1D)^33^. This is also common in video analysis where each frame (2D) has a temporal sequence. While spatial-spectral CNNs are well-suited for hyperspectral data due to the rich spectral information and high dimensionality, their application to polarization images might not yield the same benefits. Polarization SHG images primarily capture spatial patterns influenced by polarization angles, and the limited angular diversity might not warrant the use of a dedicated 1D processing component. The state-of-the-art 2D ResNet captures the intricate features from the input progressive multipolarization SHG image data and predicts the proportion values as the output. Combining intensity and polarization channels allows the network to fuse complementary information. This can result in a more holistic representation of the scene, enhancing the model's ability to capture complex patterns and structures. To date, although several studies have been conducted to automate the differentiation of collagen fibers in diseases using SHG images, the underlying variation in polarization images has not been well explored. Therefore, this study has the potential to explore the automation of polarization dependent SHG imaging to advance the conventional pixel resolution-based analysis methods. In conclusion, although there is potential in integrating multipolarization SHG images into this ResNet-based regression network, its current ability to differentiate collagen blends may benefit from further optimization.

## Conclusions

The limitations observed in the pixel-wise analysis emphasize the need for an automated method that can effectively eliminate human intervention and simplify parameter extraction processes, especially when dealing with pure collagen gel types. The proposed multichannel 2D ResNet regression model based on the multipolarization SHG imaging introduces a potential approach for the automated discrimination of collagen blend hydrogel, offering a more efficient and rapid analysis process compared to conventional methods. The utilization of the ResNet model, trained on the multipolarization SHG images at different intervals, showcased a remarkable categorization performance as compared to the model trained on the nonpolarization SHG images. Given time limitations and the primary objective of showcasing the feature extraction potential from multipolarization SHG images, the exploration of combining 3D spatial information with multipolarization SHG images has not been pursued in this study. Overall, the integration of the multipolarization SHG images and the deep learning technique presents a promising avenue for further research in collagen analysis.

## Materials and methods

### Collagen type I and II blend hydrogel preparation

Fiber samples are prepared in vitro using collagen type I sourced from rat tail (354249, Corning, USA) and collagen type II sourced from bovine articular cartilage (354257, Corning, USA). Each set of SHG signature comparisons between the two collagen types is conducted with gels polymerized simultaneously to ensure consistent conditions. To ensure sufficient sample size, the experiment is repeated three times for replicates. A working concentration of 1 mg/mL for both collagen types is prepared on ice, and the total gel volume is 0.3 mL, diluted with a phosphate-buffered saline (PBS) (Gibco™, 10010023) solution. To neutralize the pH of the collagen solution, a sterilized sodium hydroxide (NaOH) (Sigma-Aldrich, 1310–73-2) solution is used. The collagen solution is then slowly polymerized in a micro-slide 8-well culture chamber, and maintained at 4 °C for 20 h. The resulting gels, with a thickness of approximately 1.5 mm, are gently released from the bottom of the dish and subsequently fixed in 4% paraformaldehyde (Sigma-Aldrich, 158127) for further analysis. Similarly, all the collagen type I and II blend hydrogels are prepared with 0%, 25%, 50%, 75%, and 100% type II, respectively. Additionally, a few more collagen type I and II blend hydrogels at 0%, 20%, 40%, 60%, 80%, and 100% type II are also prepared to infer the untrained images. The abovementioned procedures of polymerization of collagen fibers are repeated as it is in the case of collagen blend samples.

### Overall multipolarization SHG imaging system with network

**Figure 4:**
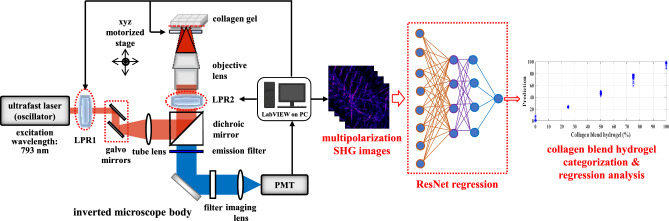
Left: schematic representation of dual-liquid crystal polarization based SHG imaging. Right: 2D ResNet regression with multichannel for categorization and regression analysis of collagen blend hydrogel.

The multipolarization SHG imaging setup employed in this study is based on a well-established optical system, as detailed in a prior publication^11^. Briefly, the system consists of an inverted microscope (Axiovert 200, Zeiss Germany) with a mode-locked Ti:sapphire laser (Tsunami, Spectra-Physics, USA) serving as the excitation source (see Fig. 4). The experiments are conducted using an excitation wavelength of 793 nm, with an average power of 10 mW at the sample plane. The laser pulses have a width of 150 fs, and the repetition rate is set at 80 MHz. To control the laser power at the focal plane, a combination of a half-wave plate and a linear polarizer was utilized. For image acquisition, the system employs a field size of 50 × 50 μm^2^, a 256 × 256 pixel, and an exposure time of 1 s. The P-SHG signal is collected in the backward direction using a 390 nm ± 20 nm bandpass filter (BWSHG: HQ390/20, Chroma Technology) and a 20X, 0.7 NA objective lens (UPlanApo, Olympus). Key components of the setup include two galvanometer *x* and *y* scanners (6215H, Cambridge, USA), a *z*-axis piezoelectric nano-positioning stage (Nano-F100, Mad City Labs, USA), and a photomultiplier tube (PMT) (H5783P, Hamamatsu, Japan).

To investigate collagen structural data under different polarizations, the setup incorporates two liquid crystal polarization rotators (LPRs) (Meadowlark Optics, USA). Linear polarizations are achieved by precisely rotating the first LPR (as LPR1 in Fig. 4) to the desired angle through voltage control. To generate left-hand circular polarization and right-hand circular polarization lights, the second LPR (i.e., LPR2) is positioned outside the microscope body. The polarization calibration is performed using a tendon sample to check the response profile of intensity as a function of polarization angle. However, calibration is not verified for every individual experiment, which could potentially lead to slight shifts in one or two polarizations. The multipolarization SHG images incorporated multichannel 2D ResNet chosen for training and inference, is illustrated on the right of Fig. 4. More details on the ResNet architecture used in this study will be presented in the following section.

### The 2D ResNet regression with multipolarization SHG images

**Figure 5:**
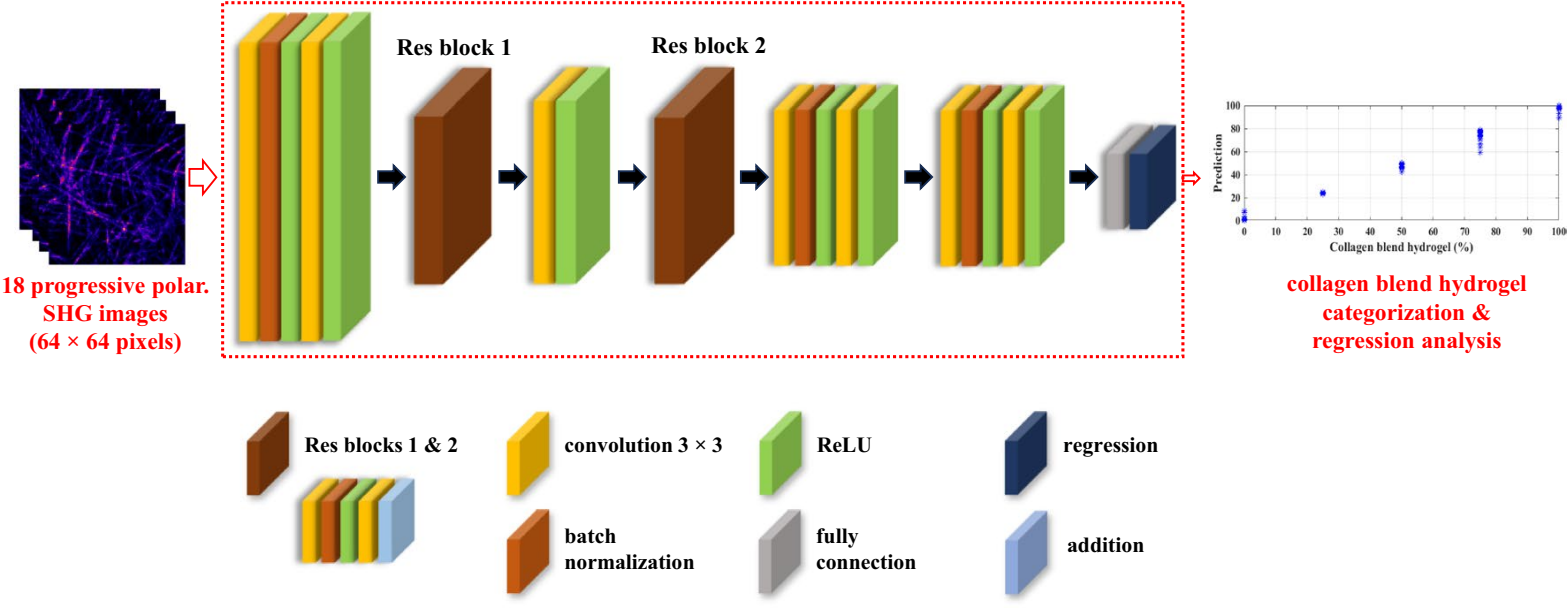
The 2D ResNet regression architecture with 18 progressive polarization SHG images for different collagen blend categorization and regression analysis.

A comparative analysis is performed between the two variations of the 2D ResNet model. One variant of 2D ResNet utilizes the nonpolarization SHG images, while the other incorporates multipolarization SHG images presented in a multichannel format. Multi-modality images provide a more comprehensive view of the real world by combining different imaging modalities. This allows for the extraction of various types of information such as thermal radiation differences, texture details, intensity and polarization information, and spatial, spectral, and temporal information^34^. Typically, data analysis for discriminating different collagen types stands on the P-SHG information at the pixel level, whereas DL methods integrate data at the feature level. Here, Fig. 5 presents a modified multichannel 2D ResNet architecture designed for regression tasks, focusing on predicting continuous values rather than class labels. The dataset consists of multipolarization SHG images as input channels and corresponding proportion values as ground truth. The training model is implemented under the MATLAB Deep Learning Toolbox in a single NVIDA GeForce RTX 3090. Before training, the dataset containing 100 sets of multipolarization SHG images is split into training and validation sets. The ratio between the training and validation sets was 3:1. The training and validation data are further normalized by dividing by 100 to bring the concentration values into the range of [0, 1]. During DL model training, a data augmentation technique is used to improve predictive performance. This approach involves dividing the larger images into smaller overlapping patches of size 64 × 64 pixels with an overlap of 50%. Moreover, a 5-degree rotation interval is also introduced, generating around 148,000 multipolarization SHG images and 135,000 nonpolarization SHG images. The network architecture comprises several layers, including convolutional layers, batch normalization layers, ReLU activation functions, and residual connections. The network layers start with a grouped convolution 2D layer with 16 filters applied separately for channel-wise convolution, followed by batch normalization and ReLU activation. This layer enables the network to process multiple input polarization channels effectively. Additional layers include convolutional layers, batch normalization layers, ReLU activation functions, and residual connections. Two residual connections are introduced to facilitate learning and improve gradient flow in the network. The final layers include a fully connected layer and a scaling layer to further scale the output to the desired range [0, 1]. The output layer utilizes a regression layer to perform the regression task for predicting collagen blend concentration proportion values. The combination of channel-wise processing and skip connections enables the network to capture and leverage angle-specific features for accurate regression tasks. Training options, such as the optimizer (Adam), learning rate, number of epochs, and mini-batch size, are set. The defined network values include the learning rate set to 1e-4, the patch size for training images set to 64, and the maximum number of training epochs set to 500. The network's performance is evaluated using metrics like root mean square errors and loss for training and validation. The training and validation performance are visualized using plots to track the model's learning progress. Following training, the trained network is assessed on the validation set, and predictions for the proportion of collagen blend values are obtained. The code then calculates the mean and standard deviation of the predictions for different concentration levels. Various statistical measures, including mean concentration, median concentration, and standard deviation, are computed for the predicted concentrations of each image. The model's accuracy in predicting concentrations is evaluated through visualization and analysis of prediction errors. The MAE is computed by calculating the mean difference between the predicted and actual values across all the collagen type I and II blend hydrogels for both the models trained on nonpolarization images and multipolarization images. The inference execution time is around 4.05 s for the multipolarization images and about 3.51 s for the nonpolarization images.

## Data Availability

All of the data generated and analyzed during this study are included in this article.
